# Intuitive Eating Behavior, Diet Quality and Metabolic Health in the Postpartum in Women with Gestational Diabetes

**DOI:** 10.3390/nu14204272

**Published:** 2022-10-13

**Authors:** Dan Yedu Quansah, Sybille Schenk, Leah Gilbert, Amar Arhab, Justine Gross, Pedro-Manuel Marques-Vidal, Elena Gonzalez Rodriguez, Didier Hans, Antje Horsch, Jardena J. Puder

**Affiliations:** 1 Obstetric Service, Department Woman-Mother-Child, Lausanne University Hospital and University of Lausanne, 1011 Lausanne, Switzerland; 2 Service of Endocrinology, Diabetology and Metabolism, Department of Medicine, Lausanne University Hospital and University of Lausanne, 1005 Lausanne, Switzerland; 3 Department of Medicine, Internal Medicine, Lausanne University Hospital and University of Lausanne, 1011 Lausanne, Switzerland; 4 Interdisciplinary Center of Bone Diseases, Bone & Joint Department, Lausanne University Hospital and University of Lausanne, 1011 Lausanne, Switzerland; 5 Institute of Higher Education and Research in Healthcare (IUFRS), University of Lausanne, 1010 Lausanne, Switzerland; 6 Neonatology Service, Department Woman-Mother-Child, Lausanne University Hospital and University of Lausanne, 1011 Lausanne, Switzerland

**Keywords:** intuitive eating, diet quality, dietary adherence, metabolic health, postpartum, insulin resistance

## Abstract

Little is known regarding intuitive eating (IE), diet quality and adherence. We investigated the associations between IE, diet quality and metabolic health after gestational diabetes (GDM), who have an increased diabetes risk. Data from 179 women with GDM from MySweetheart trial (NCT02872974) were analyzed. IE was assessed using the eating for physical rather than emotional reasons (EPR) and reliance on hunger and satiety cues (RHSC) subscales of the French Intuitive Eating Scale-2. Metabolic outcomes included weight, central body fat and insulin resistance. Diet quality was calculated using the Alternative Health Eating Index (AHEI) and compliance with national recommendations was evaluated. Both IE subscales were associated with lower BMI and fat mass (BIA) at 1-year postpartum (all *p* ≤ 0.034). The EPR subscale inversely correlated with fat mass (DXA) and visceral adipose tissue (both *p* ≤ 0.028), whereas RHSC with higher insulin sensitivity (Matsuda, *p* = 0.034). RHSC during pregnancy predicted increased AHEI (*p* = 0.043) at 1-year postpartum, whilst EPR predicted lower fat mass and insulin resistance (HOMA-IR) (all *p* ≤ 0.04). In longitudinal analyses, both subscales were associated with increased adherence to dairy and fiber intake recommendations (both *p* ≤ 0.023). These data suggest IE may be an interesting approach to improve diet quality and metabolic outcomes in women with GDM.

## 1. Introduction 

Eating behavior is linked to food choices, eating practices, dieting, and eating-related problems. Intuitive Eating (IE) is an adaptive and regulated eating behavior that controls emotional eating and dietary restriction [1]. It is a non-dieting approach, which deals with the ability to interpret and adhere to instinctive feedback regarding the amount and timing of food intake. IE has three distinct characteristics: (1) reliance on internal hunger and satiety cues to determine when to start and stop eating; (2) nonrestrictive eating, and (3) eating for physical rather than emotional reasons [2,3].

Diet quality refers to a dietary pattern or an indicator of food variety across key food groups recommended in dietary guidelines [4]. The Alternate Healthy Eating Index (AHEI) is a quantitative score measuring diet quality [5]. An updated review of 34 observational cohorts revealed that higher dietary quality is associated with adherence to dietary recommendations and lower all-cause mortality, metabolic and cardiovascular risks [6]. Diet quality can influence morbidity and mortality beyond weight (or glucose control) and both quality and adherence have long-lasting benefits for metabolic health [6,7].

IE can potentially improve dietary adherence, and encourage self-responsibility with food [1]. Although cross-sectional studies outside of pregnancy found no association between IE and diet quality [8,9], an IE intervention study in the general population led to an improved diet quality [10]. In pregnancy, one cross-sectional study suggested an association between IE and diet quality in the second and third trimesters of pregnancy [11], but longitudinal studies and studies in the postpartum are lacking. We are not aware of studies relating IE to adherence with national or international dietary recommendations.

Relationships between IE and body mass index (BMI), weight, gestational weight gain [12,13] in the general pregnant population and with weight [14] in the postpartum have been reported. We previously also showed both cross-sectional and longitudinal associations between IE with weight, BMI, weight retention and glucose control in the perinatal period up to 1-year postpartum in metabolically high-risk women with gestational diabetes (GDM; women with a glucose intolerance diagnosed in pregnancy) [15,16]. Data on IE with body composition including total and central body fat, and insulin resistance, both of which are correlates of adverse metabolic health outcomes in the postpartum period in women with GDM, are lacking. The perinatal period is an essential moment regarding maternal metabolic health. Existing recommendations aimed to improve metabolic health include women returning to their pre-pregnancy weight or a 5% decrease in weight if they are overweight or obese. However, at least two-thirds of women do not even return to pre-pregnancy weight at 1-year postpartum [17,18]. In women with GDM, postpartum weight is the most important predictor for future diabetes [19]. In view of the 7-fold increased risk for diabetes and cardiovascular outcomes [20,21], the postpartum period is especially a critical moment for women with GDM. Data on the relationship between IE, diet quality and metabolic health are lacking, and it may be promising to explore the potential benefits of IE as an alternative approach to promote healthy eating and to prevent short and long-term adverse metabolic health outcomes in the postpartum period in women after GDM.

We investigated the cross-sectional and longitudinal associations between IE during and after pregnancy with diet quality and metabolic health including weight, total and central body fat, and insulin resistance at 1-year postpartum in women with GDM. We also explored if a higher IE score during pregnancy was related with higher adherence with the Swiss Society of Nutrition (SSN) dietary recommendations at 1-year postpartum.

## 2. Methods

### 2.1. Study Design and Patient Population

**Figure 1:**
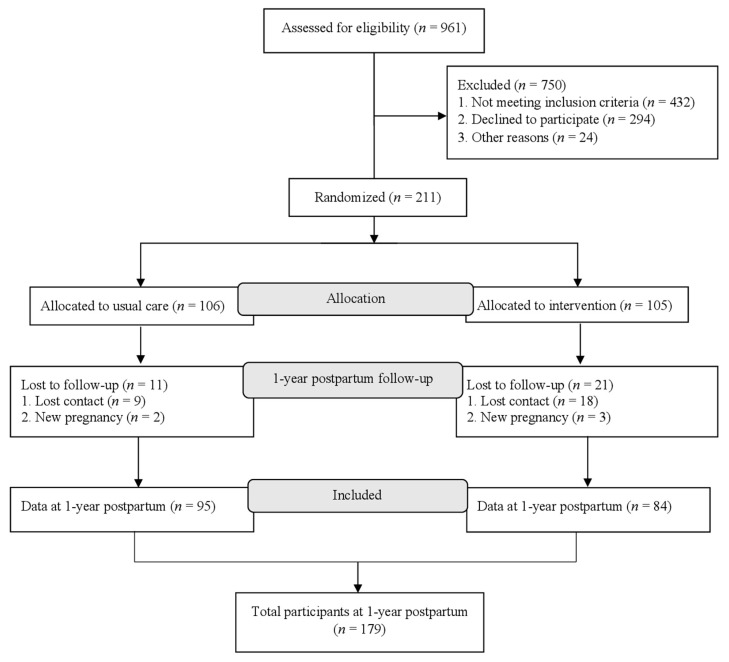
Flow of study participants. Of the 211 women included at baseline, 179 completed the 1-year follow-up and were included in this analysis. Out of these 179 women with data at 1-year postpartum visit, 100 had valid dietary data and 162 had valid data for HOMA-IR.

This current study is based on data from the MySweetheart trial (trial registration: NCT02872974) data. The detailed study protocol has been previously described [22]. Briefly, the MySweetheart trial tested the effect of an interdisciplinary lifestyle and psychosocial intervention on improving metabolic and mental health outcomes in women with GDM up to 1-year postpartum [22]. General eligibility criteria included women ≥18 years, diagnosed with GDM between 24–32 weeks gestational age (GA) according to the International Association of Diabetes and Pregnancy Study Groups (IADPSG) and the American Diabetes Association (ADA) guidelines [23,24]. Of the 211 participants included at baseline (105 randomized to intervention and 106 to usual care, Table 1), 179 completed the 1-year postpartum follow-up (Figure 1) and were included in the analyses. Predictors and outcomes of interest in this analysis were similar in the intervention and usual care groups so we pooled all participants together and adjusted for group allocation (see statistical analysis).

### 2.2. GDM Management and Patient Follow-Up

Women in the usual care group were followed-up according to the current ADA and Endocrine Society guidelines [23,25]. Following the diagnosis of GDM, women were seen at 24–32 weeks GA by either a physician, or a diabetes-specialist nurse, and were followed until childbirth. During this visit, they received information on GDM, specific recommendations regarding lifestyle changes and gestational weight gain (GWG) based on the 2009 recommendations of the Institute of Medicine (IOM) [26]. We placed a strong focus on lifestyle behavioral changes. Women were taught how to perform self-control of blood glucose both fasting and 2 h postprandial. Women also had one appointment with a registered dietician in order to receive individualized dietary advice, which focused on distribution of carbohydrate intake over several meals and snacks, limiting the intake of free sugars to less than 10%, and increase fiber intake to up to 30 g per day [27]. Women were advised to reduce sedentary behavior and engage in physical activity ideally postprandial. Treatment with insulin, or rarely with metformin was introduced when glucose values remained above targets between two or more times during a 1 to 2-week period (fasting plasma glucose) (FPG) > 5.3 mmol/l, 1-h postprandial glucose > 8 mmol/l and/or 2-h postprandial glucose > 7 mmol/l) despite lifestyle changes according to Swiss guidelines [28]. At 1-year postpartum, patients underwent a 75 g oral glucose tolerance test (oGTT) and received general advice on lifestyle changes.

On top of the usual care, the intervention consisted of four clinical lifestyle sessions during pregnancy and four lifestyle and psychosocial visits in the postpartum, two peer support group workshop (one in pregnancy and one in the postpartum), and a bimonthly lifestyle coach support, mostly through telemedicine. It focused on tailored behavioral and psychosocial strategies to improve diet, physical activity, mental health and social support, and to improve adherence to GWG and weight retention recommendations. Of the four additional sessions during pregnancy, women had two counseling sessions with a dietician that encouraged mindful eating, and prioritized higher quality fats, reducing the intake of red or processed meat or high-fat cheese. They also had two additional sessions with the physiotherapists to increase physical activity. In the postpartum, continuous breastfeeding for at least 6 months was encouraged and aerobic and resistance physical activity for 150 min a week and resistance physical activity twice a week, all at a moderate intensity, were recommended during one of the postpartum visits.

### 2.3. Measures

All outcome measures including IE, metabolic health (except those assessed at only 1-year; see below) and dietary variables including diet quality were assessed both at the first GDM visit during pregnancy (baseline) and at 1-year postpartum.

#### 2.3.1. Baseline Demographic and Health Characteristics

Data on maternal socio-demographic characteristics including age, nationality/ethnic origin and educational level were collected during the first GDM visit. Information on medical characteristics including previous history of GDM, family history of diabetes, gravida, parity and family social support during pregnancy (living with partner or with support, yes/no) were extracted from participants’ medical charts.

#### 2.3.2. Intuitive Eating Assessment 

We assessed IE with a 14-item self-report questionnaire consisting of “eating for physical rather than emotional reasons” (EPR, 8 items) and the “reliance on hunger and satiety cues” (RHSC, 6 items) subscales of the French Intuitive Eating Scale-2 (IES-2) [29]. Originally, the French IES-2 contains three (3) subscales including the EPR, the RHSC and the unconditional permission to eat (UPE) subscale. Although we measured IE before the diet visit, we did not include the UPE subscale in this study, because the diagnosis of GDM itself and subsequent dietary counselling could significantly influence responses to the UPE subscale questions, such as “I try to avoid certain foods high in fat, carbohydrates, or calories”. However, we measured metabolic health and dietary intakes before the dietician visit to ensure that, diet counselling did not influence study outcomes.

Women completed the EPR and RHSC subscales by responding to a 5-point Likert scale response ranging from one (“strongly disagree”) to five (“strongly agree”) to each item in both subscales. Possible scores for each subscale ranged from 1 to 5. We then calculated the EPR and RHSC subscale scores as recommended [29]. A higher score of the EPR subscale reflects eating as an answer to hunger and a lower score meant eating to cope with emotional distress, whereas a higher score of the RHSC subscale signifies trust in internal cues, and a lower score reflects less ability to regulate food intake.

#### 2.3.3. Dietary Intake, Diet Quality (AHEI) and Dietary Adherence

Dietary intake was assessed with a validated food-frequency questionnaire (FFQ) for French-speaking Swiss adults. Details of this FFQ are described elsewhere [30]. This FFQ assesses the intakes of 97 different food items in the previous 4 weeks. For each food item, consumption frequencies ranged from “less than once during the last 4 weeks” to “2 or more times per day”. We compared participants’ average portion sizes (smaller, equal or bigger) to a reference size. Reported consumption frequencies were converted into daily or weekly consumptions including: “never these last 4 weeks” = 0; “once/month” = 1/28; “2–3/month” = 2.5/28; “1–2/week” = 1.5/7; “3–4times/week” = 3.5/7; “once/day” = 1 and “2+/day” = 2.5. We summed all frequencies of foods in a food category to obtain the consumption frequency of that food category. Conversion into food nutrients were based on the French CIQUAL food composition table. We expressed total protein, carbohydrate and fat as percentage of total energy intake (TEI) (alcohol excluded).

We calculated Alternative Health Eating Index (AHEI) as a measure of diet quality. The AHEI was adapted from McCullough et al. [5]. In our study, we could not assess the amount of trans fat. The modified AHEI score ranged between 2.5 and 77.5 instead of 2.5 and 87.5 for the original AHEI score [5]. Higher AHEI scores represented a higher diet quality and a healthier diet. At 1-year postpartum, compliance with the national Swiss Society of Nutrition (SSN) dietary recommendations were computed as recommended [31]. The recommendations include ≥2 fruit portions/day; ≥3 vegetable portions/day; ≤5 portions meat/week; ≥1 portion fish/week and ≥3 portions dairy products/day and fiber ≥30 g/day [32]. For each recommendation, a binary variable (1 = yes, 0 = no) was computed.

#### 2.3.4. Metabolic Health Outcomes

Pre-pregnancy weight was extracted from participants’ medical charts or, if missing, was self-reported. We measured height at the first GDM visit during pregnancy and weight at the same moment and at 1-year postpartum to the nearest 0.1 cm and 0.1 kg with electronic scales (Seca® model 220, Hamburg, Germany). BMI was expressed as a ratio of weight in kilograms to the square of height in meters (kg/m^2^). Total GWG during pregnancy was defined as the difference in pre-pregnancy weight and weight at the end of pregnancy. Fat mass was estimated using Kyle equation based on reactance and resistance values from Bioelectrical Impedance Analysis (BIA) (Akern BIA 101, Akern Srl, Pontassieve, Italy) measures performed during the first visit and at 1-year postpartum [33]. We also assessed fat mass and visceral adipose tissue at 1-year postpartum using Dual-Energy X-ray Absorptiometry (DXA; iDXA device, GEHC-Lunar, Madison, WI, USA) in 109 women who signed an additional consent form for this procedure.

We extracted data on the need for glucose-lowering medical treatment during pregnancy (use of insulin and/or metformin) from maternal medical records. We measured fasting glucose, HbA1c and calculated Homeostatic Model Assessment for Insulin Resistance (HOMA-IR) at the first GDM visit. At 1-year postpartum, women underwent a 75 g oGTT with glucose and insulin sampling at 30 min intervals for 2 h. We calculated insulin resistance/sensitivity, both by HOMA-IR (mostly hepatic insulin resistance) and MATSUDA (mostly total body insulin sensitivity) [34,35].

### 2.4. Statistical Analyses

All statistical analyses were performed with Stata/SE 15.1 (StataCorp LLC, College Station, TX, USA) [36]. We presented demographic and other descriptive variables as means (±standard deviation) or percentages (%) where appropriate. Predictors (EPR and RHSC subscales) and outcomes including diet quality (AHEI), all metabolic health variables (weight, BMI, GWG, total fat mass (BIA and DXA), visceral adipose tissue (DXA) and measures of insulin resistance (HOMA-IR, MATSUDA)) were normally distributed. We used a paired *t*-test to determine the changes in EPR and RHSC, AHEI and metabolic health variables between the first GDM visit and the 1-year postpartum. We performed linear regression analyses to determine the associations between EPR and RHSC with AHEI and metabolic health, namely the cross-sectional association between EPR and RHSC with AHEI and metabolic health at 1-year postpartum, and longitudinal associations between EPR and RHSC at the first GDM visit during pregnancy with AHEI and metabolic health at 1-year postpartum.

We used a modified Poisson regression for dichotomous variables to determine the longitudinal relationship between EPR and RHSC at the first GDM visit during pregnancy and adherence to SSN dietary recommendations for fruits, vegetables, dairy, protein and fiber intakes at 1-year postpartum. In all analyses, predictors and outcomes were similar in both groups (intervention vs. usual care), and the results, particularly the effect sizes, were similar when the regression analyses were restricted only to the usual care group or both groups together. Therefore, and to increase the sample size, we pooled both groups and adjusted for group allocation in all analyses. All reported beta-coefficients for all regression estimates were standardized. All statistical significances were two-sided and accepted at *p* < 0.05.

## 3. Results

### 3.1. Baseline Characteristics of Participants

Among the 211 women included at the first GDM visit, 179 (84.8%) completed the 1-year follow-up visit (Figure 1). Mean gestational age, pre-pregnancy weight and BMI at baseline were 29.0 ± 2.2 weeks, 69.0 ± 14.8 kg and 25.6 ± 5.1 kg/m^2^ respectively (Table 1).

Table 2 shows the change in IE, diet quality and metabolic health variables between the first GDM visit and 1-year postpartum. Whereas the EPR subscale remained stable (0.01 ± 0.8), the RHSC increased by 0.13 ± 0.9 at 1-year postpartum (*p* = 0.041). Nationality/ethnic origin of women did not influence EPR and RHSC scores (data not shown). Dietary intake variables including AHEI scores (Table 2), TEI, total carbohydrate, protein, fat and fiber intakes (results not shown) were similar at both time points (all *p* ≥ 0.07), whereas all measures of metabolic health including BMI, weight, fat mass (BIA), and insulin resistance (HOMA-IR), significantly decreased at 1-year postpartum (all *p* ≤ 0.007).

### 3.2. Cross-Sectional Associations between IE, Diet Quality and Metabolic Health

In the cross-sectional analyses at 1-year postpartum, none of the subscales of IE was associated with AHEI (Table 3). Similarly, they were not associated with HOMA-IR. However, they were inversely associated with lower BMI (EPR: β = −1.17, 95% CI:−2.13, −0.21; RHSC: β = −1.20, 95% CI:−2.14, −0.22,) and fat mass (EPR: β = −2.01, 95% CI:−3.85, −0.17; RHSC: β = −2.02, 95% CI:−3.97, −0.08). Additionally, the EPR subscale correlated with lower weight (β = −2.82, 95% CI:−5.59, −0.04), fat mass (DXA) (β = −4.44, 95% CI:−7.08, −1.80) and visceral adipose tissue (β = −0.12, 95% CI:−0.23, −0.02) whereas the RHSC was associated with higher total body insulin sensitivity (MATSUDA, β = 0.18, 95% CI: 0.006, 0.128).

### 3.3. Prospective Associations between IE, Diet Quality, Metabolic Health and Dietary Adherence

In the longitudinal analyses (Table 4), the EPR subscale at the first GDM visit was associated with a decreased BMI (β = −1.42, 95% CI:−4.21, −0.03), fat mass (DXA) (β = −2.95, 95% CI:−5.95, −0.02) and lower insulin resistance (HOMA-IR; β = −1.61, 95% CI:−3.21, −0.09) at 1-year postpartum but not with AHEI. On the other hand, the RHSC subscale during pregnancy predicted an improved AHEI score (β = 2.03, 95% CI: 0.09, 3.97) at 1-year postpartum. RHSC was also associated with both lower GWG during pregnancy and weight at 1-year postpartum (both *p* ≤ 0.041).

We also investigated the relationship between IE at the first GDM visit and adherence to the SSN dietary recommendations for adults at 1-year postpartum (Table 5). Both subscales predicted an increased adherence to dairy and fiber intakes recommendations (*p* ≤ 0.023). Additionally, the RHSC subscale was associated with an increased adherence to the fruit intake recommendation (*p* = 0.024).

## 4. Discussion

This study found cross-sectional and longitudinal associations between intuitive eating (IE) with increased diet quality, healthier metabolic outcomes and increased adherence to dietary recommendations in women after GDM. Specifically, the cross-sectional analyses at 1-year postpartum showed an inverse association between the two subscales of IE (eating for physical rather than emotional (EPR) and reliance on hunger and satiety cues (RHSC) subscales) with BMI and fat mass (BIA). Additionally, the EPR subscale correlated with lower fat mass (DXA) and visceral adipose tissue, whereas the RHSC was associated with higher total body insulin sensitivity (MATSUDA). In the longitudinal analyses, RHSC in pregnancy predicted increased dietary quality (AHEI scores) and lower weight at 1-year postpartum, whilst EPR was inversely associated with BMI, fat mass (DXA) and insulin resistance (HOMA-IR). Higher scores of both subscales predicted an increased adherence to dairy and fiber intake and RHSC further predicted fruit intake recommendations at 1-year postpartum. These results shed new light on the importance of IE for nutrition and metabolic health in the perinatal period in these women.

Although the postpartum period is a critical and important public health period for metabolic health, particularly in women with GDM, there has been a lack of studies of IE and diet quality. Furthermore, there is a general need for longitudinal studies regarding IE. The longitudinal relationship between RHSC and increased AHEI scores in our study suggests that IE can promote diet quality [2,3]. IE emphasizes/focuses on internal cues of hunger and satiety, which are linked to diet quality by developing a positive relationship with food and instinctively recognizing food variety to promote nutritional balance [8]. Our results are concordant with a cross-sectional study that showed positive association between IE and diet quality during pregnancy in a general pregnant population [11]. The findings of this study are in agreement with several studies performed outside of the perinatal setting. This includes an IE intervention that increased diet quality [10], an IE program that reduced restrictive dieting and emotional eating towards a healthy eating approach, ultimately promoting better diet quality [37] and a Swiss study that reported positive associations between EPR and RHSC with diet quality in women [38].

IE principles including honoring hunger and health [39] overlap with the key concepts of dietary adherence including making healthier food choices, healthy eating patterns and food variety [40]. It is therefore not surprising that higher scores of EPR and RHSC subscales during pregnancy predicted increased adherence to dairy and fiber intake recommendations at 1-year postpartum in our study. The latter also predicted an increased adherence to fruit recommendations. The relationship between IE practices and self-responsibility with food [1] leads to appropriate consumption of diverse food groups that influence dietary adherence [41]. Studies in the general population that showed associations between EPR subscale with lower intakes of dairy, meat and fish [12], lower intakes of sweets and fast foods [38] and with higher intakes of fruits, vegetables and whole grains [8] are in general consistent with our results. However, in contrast to our study, these studies rather demonstrate a relationship with healthy food choices, but do not investigate the relationship between IE and adherence to dietary recommendation of (inter) national scientific organizations. Adherence to dietary recommendations is an important predictor of long-term weight loss and improved metabolic outcomes [42].

In this study, the RHSC subscale predicted lower weight, whereas the EPR was inversely associated with BMI, fat mass (DXA) and insulin resistance (HOMA-IR). Increased IE practices reduce emotional eating triggers (anxiety and depression) that are often associated with increased weight gain [43] and its associated metabolic problems that are accentuated in women with a history of GDM. We also speculate that a significant positive relationship between IE with diet quality and adherence might play a role, as both diet quality and adherence have short- and long-term metabolic benefits [44].

Our finding of cross-sectional associations between the two subscales of IE and lower weight and BMI at 1-year postpartum in women after GDM has been previously reported by our research group [15,16]. However, this is the first to investigate and show the inverse association between IE with fat mass, visceral adipose tissue and with higher total body insulin sensitivity. Both visceral adipose tissue and insulin sensitivity have important clinical implications and are independently associated with cardio-metabolic outcomes in women after GDM [45]. The cross-sectional and longitudinal data of our study show the relationship between IE with central body fat and insulin resistance in women with GDM.

The strengths of this study include its design and longitudinal follow-up. It is the first study to investigate the relationship between IE with diet quality and adherence to dietary recommendations, particularly in women with GDM. It is also the first to show an association between IE with lower weight and metabolic parameters in the postpartum, notably fat mass and its distribution. Limitations include the use of self-reported data regarding IE and dietary intakes. Despite being self-reported, we used validated tools to measure IE, diet quality and compliance with SSN dietary recommendations. Another potential limitation is the lack of total IES-2 scores, as we did not include the UPE subscale because of the potential response bias described above. At 1-year postpartum, 15% (*n* = 32/211) of women were lost to follow-up, but this proportion is lower compared to other studies in women with GDM.

In the future, a more objective outcome measure for IE would be helpful. In addition, future research that utilizes IE intervention in women with GDM, starting early in pregnancy up to 1-year postpartum to increase diet quality, improve dietary adherence and metabolic outcomes both in pregnancy and in the postpartum are needed to determine the cause and effect of our associations.

## 5. Conclusions

We found cross-sectional and longitudinal associations between IE with increased diet quality (AHEI) and healthier metabolic outcomes at 1-year postpartum in women after GDM. Thus, the subscales of IES-2, RHSC and/or EPR, inversely correlated with weight, body fat and insulin resistance. Higher scores of both subscales predicted an increased adherence to national dietary recommendations. Particularly the longitudinal associations imply that IE during and after pregnancy can be an alternative healthy eating approach that can increase diet quality, improve adherence, and reduce the risk of adverse metabolic outcomes in the postpartum in women after GDM who have an increased metabolic risk in the postpartum period.

## Figures and Tables

**Table 1:** Baseline maternal socio-demographic and health characteristics (*n* = 179).

Variable	Mean ± SD
Age (year)	33.6 ± 5.0
GA at the first GDM visit (weeks)	28.9 ± 2.2
Pre-pregnancy weight (kg)	69.0 ± 14.9
Pre-pregnancy BMI (kg/m^2^)	25.59 ± 5.1
Weight at the first GDM visit (kg)	79.32 ± 14.6
BMI at the first GDM visit (kg/m^2^)	29.41 ± 4.9
HbA1c at the first GDM visit	5.09 ± 0.3
Fasting glucose at GDM visit	4.95 ± 0.5
Ethnicity/Nationality, *n* (%)	
Switzerland	52 (28.9)
Rest of Europe and North America	68 (38.3)
Asia and Oceania	13 (7.2)
Africa	21 (11.7)
Latin America	7 (3.9)
Others	18 (10.0)
Education level ^a^, *n* (%)	
Compulsory school incomplete ^b^	2 (1.3)
Compulsory school achieved	21 (13.9)
High school	16 (10.6)
General and vocational education	32 (21.2)
University	80 (53.0)
Employment status	
Student	5 (2.8)
Professional worker	118 (65.9)
Housewife/unemployed	56 (31.3)
Glucose-lowering treatment in pregnancy, *n* (%)	
None	99 (55.6)
Insulin	70 (38.9)
Metformin	10 (5.6)
Parity, *n* (%)	
0	102 (57.2)
1	49 (27.2)
2	14 (7.8)
≥3	14 (7.8)
Gravida, *n* (%)	
1	78 (43.3)
2	40 (22.8)
≥3	61 (33.9)
GDM in previous pregnancy ^c^, yes, *n* (%)	19 (10.6)
Family history of diabetes, yes, *n* (%)	109 (61.1)
Social support during pregnancy, yes, *n* (%)	12 (6.7)

GDM denotes gestational diabetes mellitus; GA denotes gestational age; BMI denotes body mass index; ^a^ 29 participants had missing data on education; ^b^ in Switzerland, compulsory schooling lasts eleven years including kindergarten; ^c^ only for women who had at least one previous pregnancy; All data are expressed as *n*, % for categorical variables or mean (standard deviation) for continuous variables.

**Table 2:** Frequency, mean and standard deviation of study variables at baseline and end of the study.

Variable	*n*	First GDM Visit Mean ± SD	At 1-Year pp Mean ± SD	Mean Difference Mean ± SD	*p*-Value
Intuitive eating behaviors					
EPR subscale	179	3.8 ± 0.8	3.8 ± 0.9	0.01 ± 0.8	0.846
RHSC subscale	179	3.5 ± 0.8	3.6 ± 0.8	0.13 ± 0.9	**0.041**
Diet quality					
AHEI	100	31.7 ± 9.7	30.6 ± 9.5	−1.07 ± 11.0	0.395
Metabolic health variables					
BMI (kg/m^2^)	179	29.4 ± 4.9	26.8 ± 5.6	−2.52 ± 2.1	**<0.001**
Weight (kg)	179	79.3 ± 14.5	72.4 ± 16.1	−6.85 ± 5.7	**<0.001**
Total fat mass (BIA) (kg)	179	31.5 ± 9.2	26.6 ± 10.6	−4.91 ± 4.2	**<0.001**
HOMA-IR	162	3.6 ± 2.0	3.2 ± 2.3	−0.34 ± 1.5	**0.007**

GDM denotes gestational diabetes mellitus; SD denotes standard deviation; BMI denotes body mass index, EPR denotes Eating for Emotional Rather than Physical reasons of the French Intuitive eating Scale-2 (IES-2) questionnaire; RHSC denotes Reliance on Hunger and Satiety subscale of the IES-2; AHEI denotes Alternative Healthy Eating Index, BIA denotes Bioelectrical Impedance Analysis. HOMA-IR denotes Homeostatic Model Assessment for Insulin Resistance; *p*-value derived from paired *t*-test; bold *p*-values are significant (*p* < 0.05).

**Table 3:** Cross-sectional associations between the two subscales of intuitive eating with diet quality and metabolic health outcomes at 1-year postpartum.

Variable	Effect Estimate		
EPR Subscale at 1-year pp	Standardized Beta Coefficient	β (95% CI)	*p* Value
Diet quality at 1-year pp			
AHEI	0.04	0.56 (−1.38, 2.50)	0.548
Metabolic health at 1-year pp			
Weight (kg)	−0.15	−2.82 (−5.59, −0.04)	**0.040**
BMI (kg/m^2^)	−0.18	−1.17 (−2.13, −0.21)	**0.013**
Fat mass (BIA) (kg)	−0.16	−2.01 (−3.85, −0.177)	**0.027**
Fat mass (DXA) (kg)	−0.32	−4.44 (−7.08, −1.80)	**0.001**
Visceral adipose tissue (DXA) (kg)	−0.23	−0.12 (−0.23, −0.02)	**0.028**
HOMA-IR	−0.09	−0.25 (−0.68, 0.16)	0.184
MATSUDA	0.03	0.11 (−0.48, 0.71)	0.595
**RHSC Subscale at 1-year pp**			
Diet quality at 1-year pp			
AHEI	0.009	0.11 (−1.94, 2.17)	0.337
Metabolic health at 1-year pp			
Weight (kg)	−0.11	−2.30 (−5.26, 0.66)	0.109
BMI (kg/m^2^)	−0.09	−1.20 (−2.14, −0.22)	**0.023**
Fat mass (BIA) (kg)	−0.15	−2.02 (−3.97, −0.08)	**0.034**
Fat mass (DXA) (kg)	−0.16	−2.34 (−5.16, 0.47)	0.101
Visceral adipose tissue (DXA) (kg)	−0.12	−0.06 (−0.17, 0.04)	0.319
HOMA-IR	−0.10	−0.30 (−0.75, 0.14)	0.142
MATSUDA	0.18	0.64 (−0.006, 1.28)	**0.034**

pp denotes postpartum; EPR denotes Eating for Emotional Rather than Physical reasons of the French Intuitive eating Scale-2 (IES-2) questionnaire; RHSC denotes Reliance on Hunger and Satiety subscale of the IES-2; AHEI denotes Alternative Healthy Eating Index, BIA denotes Bioelectrical Impedance Analysis; BMI denotes body mass index, DXA denotes Dual-energy X-ray Absorptiometry. *p*-values are based on linear regression estimates adjusted for group allocation; bold *p*-values are significant (*p* < 0.05).

**Table 4:** Longitudinal associations between the two subscales of intuitive eating during pregnancy with diet quality and metabolic health outcomes up to 1-year postpartum.

Variable		Effect Estimate	
EPR Subscale the First GDM Visit	Standardized Beta Coefficient	β (95% CI)	*p* Value
Diet quality at 1-year pp			
AHEI	0.14	1.96 (−1.70, 5.63)	0.279
Metabolic health during pregnancy			
Total GWG during pregnancy (kg)	0.10	0.79 (−0.26, 1.86)	0.157
Metabolic health at 1-year pp			
Weight (kg)	−0.10	−2.17 (−5.12, 0.76)	0.146
BMI (kg/m^2^)	−0.11	−1.42 (−4.21, −0.03)	**0.016**
Fat mass (BIA) (kg)	−0.09	−1.30 (−3.27, 0.66)	0.193
Fat mass (DXA) (kg)	−0.18	−2.95 (−5.95, −0.02)	**0.043**
Visceral adipose tissue (DXA) (kg)	−0.13	−0.08 (−0.20, 0.03)	0.172
HOMA-IR	−0.20	−1.61 (−3.21, −0.09)	**0.032**
MATSUDA	0.04	0.16 (−0.49, 0.81)	0.640
**RHSC Subscale the First GDM Visit**			
Diet quality at 1-year pp			
AHEI	0.16	2.03 (0.09, 3.97)	**0.043**
Metabolic health during pregnancy			
Total GWG during pregnancy (kg)	−0.12	−1.42 (−4.20, −0.03)	**0.041**
Metabolic health at 1-year pp			
Weight (kg)	−0.14	−2.90 (−5.81, −0.04)	**0.025**
BMI (kg/m^2^)	−0.11	−0.81 (−1.84, 0.20)	0.124
Fat mass (BIA) (kg)	−0.14	−1.82 (−3.75, 0.10)	0.067
Fat mass (DXA) (kg)	−0.12	−1.63 (−4.20, 0.92)	0.206
Visceral adipose tissue (DXA) (kg)	−0.05	−0.02 (−0.12, 0.07)	0.538
HOMA-IR	−0.10	−0.30 (−0.74, 0.14)	0.203
MATSUDA	0.06	0.22 (−0.43, 0.87)	0.537

EPR denotes Eating for Emotional Rather than Physical reasons of the French Intuitive eating Scale-2 (IES-2) questionnaire; RHSC denotes Reliance on Hunger and Satiety subscale of the IES-2; GDM denotes gestational diabetes mellitus; AHEI denotes Alternative Healthy Eating Index, GWG denotes gestational weight gain; BIA denotes Bioelectrical Impedance Analysis; BMI denotes body mass index, DXA denotes Dual-energy X-ray Absorptiometry. *p*-values are based on linear regression estimates adjusted for group allocation; bold *p*-values are significant (*p* < 0.05).

**Table 5:** Longitudinal relationship between intuitive eating subscales during pregnancy and adherence to Swiss society for nutrition recommendation at 1-year postpartum.

Variable	Mean ± SD	β (95% CI)	*p* Value
EPR Subscale the First GDM Visit			
Fruits intake			
Below	3.75 ± 0.98	Ref	
Adhere	3.84 ± 0.78	0.09 (−0.39, 0.59)	0.699
Vegetable intake			
Below	3.75 ± 0.96	Ref	
Adhere	3.83 ± 0.76	−0.08 (−0.47, 0.32)	0.700
Dairy intake			
Above	3.79 ± 0.77	Ref	
Adhere	4.83 ± 0.28	1.03 (0.14, 1.93)	**0.023**
Protein (non-fried fish) intake			
Below	3.80 ± 0.75	Ref	
Adhere	3.96 ± 0.85	−0.14 (−0.39, 0.12)	0.288
Fiber intake			
Below	3.83 ± 0.79	Ref	
Adhere	4.25 ± 0.85	1.42 (1.07, 3.23)	**0.024**
**RHSC Subscale the First GDM Visit**			
Fruits intake			
Below	3.20 ± 0.68	Ref	
Adhere	3.58 ± 0.77	1.37 (0.42, 2.10)	**0.031**
Vegetable intake			
Below	3.53 ± 0.80	Ref	
Adhere	3.67 ± 0.91	0.14 (−0.27, 0.55)	0.505
Dairy intake			
Above	3.51 ± 0.81	Ref	
Adhere	4.24 ± 0.81	1.87 (0.56, 2.73)	**0.003**
Protein (non-fried fish) intake			
Below	3.56 ± 0.82	Ref	
Adhere	3.57 ± 0.78	0.17 (−0.25, 0.58)	0.901
Fiber intake			
Below	3.25 ± 0.60	Ref	
Adhere	3.56 ± 0.81	2.31 (1.98, 3.35)	**0.021**

EPR denotes Eating for Emotional Rather than Physical reasons of the French Intuitive eating Scale-2 (IES-2) questionnaire; RHSC denotes Reliance on Hunger and Satiety subscale of the IES-2; GDM denotes gestational diabetes mellitus; Ref denotes reference. Bold *p*-values are significant (*p* < 0.05). Analyses were adjusted for group allocation (intervention/control).

## Data Availability

The data presented in this study are available on request from the corresponding author. The data are not publicly available because it is a clinical data maintained and kept in a secure server at the Lausanne University Hospital.
